# Age at separation, residential mobility, and depressive symptoms among twins in late adolescence and young adulthood: a FinnTwin12 cohort study

**DOI:** 10.1186/s12889-024-19734-w

**Published:** 2024-08-17

**Authors:** Zhiyang Wang, Alyce M. Whipp, Marja Heinonen-Guzejev, Jaakko Kaprio

**Affiliations:** 1 Institute for Molecular Medicine Finland, Helsinki Institute of Life Science, University of Helsinki, PL 20 (Tukholmankatu 8), Helsinki, FI-00014 Finland; 2 Department of Public Health, University of Helsinki, Helsinki, Finland

**Keywords:** Twins, Residential mobility, Depressive symptom, Adolescent

## Abstract

**Background:**

Separating with close siblings and leaving the parental home at an early age represents a major life event for an adolescent (reflected by age at separation in a twin pair) and may predispose them to poor mental health. This study aims to examine the association of age at separation and residential mobility on depressive symptoms in late adolescence and young adulthood and to explore possible underlying genetic effects.

**Methods:**

Residential mobility consisted of the number and total distance of moves before age 17. Based on 3071 twins from the FinnTwin12 cohort, we used linear regression to assess the association of age at separation and residential mobility with General Behavior Inventory (GBI) scores at age 17 and in young adulthood. A higher GBI score indicated more depressive symptoms occurred. Then, the mixed model for repeated measures (MMRM) was used to visualize the scores’ trajectory and test the associations, controlling for "baseline" state. Twin analyses with a bivariate cross-lagged path model were performed between the difference in GBI scores, between cotwins, and separation status for the potential genetic influence.

**Results:**

Compared to twins separated before age 17, twins who separated later had significantly lower GBI scores at age 17 and in young adulthood. In MMRM, separation at a later age and a higher number of moves were associated with a higher GBI score in young adulthood. A small genetic effect was detected wherein GBI within-pair differences at age 17 were associated with separation status before age 22 (coefficient: 0.01).

**Conclusion:**

The study provides valid evidence about the influence of siblings and family on depressive symptoms in later adolescence and young adulthood while finding some evidence for a reverse direction effect. This suggests more caution in the interpretation of results. A strong association between residential mobility and depressive symptoms was affirmed, although further detailed research is needed.

**Supplementary Information:**

The online version contains supplementary material available at 10.1186/s12889-024-19734-w.

## Background

The global burden of major depressive disorder (MDD) is getting progressively worse since the last century [1]. High-income areas had a higher prevalence of MDD [2]. In 2019, according to the Global Burden of Diseases, the age-standardized prevalence of depressive disorder was 3851 per 100,000 people in Western Europe and was higher than the global level [3]. Data for the general US population shows the prevalence of depression in adolescents and young adults has been increasing since 2005 [4, 5]. Multiple risk factors for depression in childhood and adolescence have been identified such as low-quality education, stressful life events, and family influences [6].

During childhood, moving to a new location exposes children to greater social network loss than adults and their parents are less able to give them the attention they need during the process. Moving to a new location has been regarded as a type of negative life experience that can be associated with later depression [7, 8]. Residential instability or mobility describes those children who have an unstable life and are more frequently exposed to this deleterious experience. Numerous studies have suggested that high residential instability is linked to a larger risk of many psychological or psychiatric outcomes, including depression [7, 9–11]. However, the measure of residential mobility is often limited by recall bias from children’s or their parents’ questionnaires [9]. Longitudinal residential records from the national system could quantify the cumulative effect of mobility to enhance the assessment of the strength of this relationship [11].

In late adolescence and young adulthood, people will gradually part with their siblings and move to a new place due to various reasons. Because of the similar age and same generation, siblings spend more time together and have more mutual influence than parents. Long-term daily companionship from siblings promotes to a strong emotional connection, which has a significant impact on children’s development [12]. Supportive and beneficial sibling relationships, such as spending more time with each other, appears to be interconnected with fewer depressive symptoms and higher well-being levels [13]. Positive sibling bonding is also capable of relieving detrimental parental influences [14]. For a relatively extreme example in China, AIDS orphans who were separated from their siblings suffered a higher level of anxiety, depression, anger, and dissociation [15]. As a special case of siblings, in general, twins have a closer relationship and reciprocity with each other than non-twin siblings [16, 17]. The separation between cotwins, alternatively termed “relational shift”, breaks the intense intimacy and dependency and can be accompanied by emotional turmoil and confusion due to individuation [18]. Separation is an important challenge for twins during childhood and adolescence.

Moreover, separating with siblings/twins in adolescence or young adulthood may imply leaving from the parental home. Anxiety driven by early separation from parents has been connected to a series of negative mental health conditions such as complicated grief, more severe symptoms of depression, or later panic disorder [19–21]. Parental support acts like a “safety net” to enhance the resilience in coping with challenges and to sustain the successful transition of the child to adulthood [22, 23]. Family closeness, support, attentiveness, and other positive factors are associated with a later timing of leaving, while teenagers living with step-parents or incomplete families tend to leave early due to a lack of resources [24–26].

The age at separation between cotwins reflects the length of time that twins stay together and also probably with their parents. It is likely associated with the amount of family support, degree of mental maturity, and preparation time for twins to address the separation challenge, connecting to later mental health. On the other hand, we also assume the time of separation is when at least one of the cotwins leaves the parental home. Unlike questions about the family environment in surveys, age at separation by residential history in twins could be used as an objective measure to describe the separation time, as well as familial influence, validly with less information bias. Therefore, in this study, we hypothesize that older age at separation is a protective factor and residential mobility is a risk factor and aim to determine: 1) the association between age at separation and depressive symptoms and the sex-specific effect, 2) the association between residential mobility before age 17 and depressive symptoms in late adolescence and young adulthood, and 3) the genetic influence behind the association between age at separation and depressive symptoms, based on the FinnTwin12 twin cohort.

## Methods

This is a longitudinal twin cohort study.

### Participants

The study participants were from the FinnTwin12 cohort, which is a population-based prospective cohort among all Finnish twins born between 1983 and 1987. Participants were divided into two parts: the overall cohort and the intensive study. A total of 5184 initial twins enrolled at wave one (the overall cohort) when they reached age 11, and twins filled in the general age 14 (wave two), age 17 (wave three), and young adult (wave four) questionnaires with 92%, 75%, and 66% retention, respectively. In the intensive study, 1035 families were additionally invited when the twin was at age 14 (wave two) for psychiatric interviews, some biological samples, and additional questionnaires, and 1854 twins participated. They were invited back for new interviews and other measurements as young adults (wave four). Interviews (*n* = 1347) in young adulthood were completed for 73% of twins in the intensive study. The twins in the intensive study were mostly randomly selected from the overall epidemiology study, while enriched with twins at elevated familial risk for alcoholism [27]. There was no evidence for selection for family type, parental age, residence, twin sex, and zygosity after carefully assessing the non-responders at each stage [28]. The flowchart of the general FinnTwin12 cohort is presented in supplemental Fig. 1. The updated review of this cohort was published in 2019 [29].

### Measures

#### Age at separation

Age at separation was defined as the age that, for the first time, the twins no longer resided at the same address, with one or both twins moving to a new address as described elsewhere [30]. Reunificaton might occur sometimes after separation but was not considered in this study. Age at separation was generated from the residential records linking to the register of national Digital and Population Data Service Agency (DVV) in Finland since birth. The DVV, an official government agency, promotes digitalization, secures the availability of information, and provides various services including address services and moving notices. It updates the residential records rapidly ensuring accuracy. Separation indicated that the DVV residential records differed between cotwins. The residential records included the information on the north and east coordinates (EUREF-FIN), and the age moving in and out of each address. Then, we categorized it into four groups: separated before age 17, 17 to < 19.5, 19.5 to < 22, and age 22 or older. Subsequently, we created two binary variables for separation status (yes, no) before ages 17 and 22 based on the age at separation to show whether twins separated at that age. Twins without valid information on age at separation (44 individuals) or twin pairs who never separated by the end of the follow-up in December 2020 (13 pairs) were excluded from the analysis with age at separation.

#### Residential mobility

Residential mobility consisted of two variables: the number of moves (never, once, twice, three or more times) and the total distance of moves (in kilometers) before age 17 accumulated across all moves. Both were generated from the residential records. If twins did not have address information due to missing addresses or living abroad for any period, their total distance of moves was invalid and excluded from the corresponding analysis (301 individual twins). Overall, the mean total distance of moves before age 17 was 48.4 (SD: 153.2) kilometers, and the total distance of moves was distributed in a right skewed fashion.

#### Depressive symptoms

We used the short-version General Behavior Inventory (GBI) to evaluate the depressive symptoms among twins [31]. It is a self-reported inventory designed to identify mood-related behaviors such as depressive and manic symptoms [32]. The short-scale version has 10 items and uses a 4-point Likert scale from 0 (never) to 3 (very often) to query the occurrence of depressive symptoms. The total score ranges from 0 to 30. This reliable measure has been used in many Finnish studies including FinnTwin12 [31, 33, 34]. To validate the GBI, we compared it to a DSM-IV diagnosis of MDD assessed by the Semi-Structured Assessment for the Genetics of Alcoholism from the intensive study [35]. In a logistic regression analysis based on 1232 individual twins (mean age: 22.4), the GBI score in young adulthood strongly predicted MDD, with an area under the receiver operating characteristic curve (AUC) of 0.83. The GBI was assessed at age 17 and in young adulthood. We also calculated the change of GBI score between the two stages.

Another two variables for the within-pair differences in GBI score were created at both stages. The variables were constructed by subtracting the GBI score of one cotwin from the score of the other cotwin, and then we took the absolute values. Higher values indicated a higher level of within-pair difference.

#### Covariates

There were seven covariates defined a priori: sex (male, female), zygosity (monozygotic (MZ), dizygotic (DZ), unknown), smoking (never, quit, occasional, current) in young adulthood, work status (full time, part-time, irregular, not working) in young adulthood*,* secondary level school (senior high school, vocational, none) in young adulthood, paternal education, maternal education and age when twins provided the GBI assessment in young adulthood. Sex was assigned at birth because of biological dimensions. Paternal and maternal education (missing, low, medium, high) was reported by parents in wave one. These covariates were selected due to their relationship to either age at separation, mental health, or both, according to previous literature [30, 36, 37]. Additionally, the mean age when twins provided the GBI assessment in young adulthood was 24.18 years (SD: 1.68), which was only adjusted for in the analysis involving GBI in young adulthood.

### Statistical analysis

A total of 3071 individual twins was included in analyses at final, of whom 2769 provided data at the age 17 survey and 3045 in young adulthood. Because of the differential missing information pattern, sample sizes in different analyses were slightly different.

Due to the skewness of the GBI scores at both ages, we added one to the GBI score and log-transformed it. Then, linear regression was used to examine the association of age at separation and residential mobility with log-transformed GBI scores at ages 17 and in young adulthood and change of GBI score. The pre-specified covariates were adjusted in the models. We used the univariate analysis of variance to determine the statistical differences between the mean GBI score for the groups for categorical covariates. Additionally, the separation before age 17 (no/no information/reared apart, yes) was further adjusted for in the analysis with residential mobility.

Further, since we had two measures of GBI score, the mixed model for repeated measures (MMRM) was chosen to visualize the trajectory of GBI score by age at separation for twins at age 17 and in young adulthood. The MMRM method is a type of direct-likelihood analysis and uses longitudinal data to estimate the covariance between data from different time points [38]. This technique is able to consider the influence on the outcome (GBI in young adulthood) by the exposure of interest (fixed effect) and “baseline” GBI at age 17 (random effect) [39]. It also allowed us to handle the unevenness of missing information at different ages to increase the power. Covariates were adjusted for. Stratification analyses by sex were also performed, given that depression is more common in females than in males after puberty.

To evaluate the potential genetic influence between separation and depressive symptoms, intraclass correlation coefficients (r) were calculated at first, as an exploration. This was done to decompose overall variance into the genetic variances: additive genetic (A) and dominant genetic (D) components and environmental variances: common environmental (C), and unique environmental (E) components for depressive symptoms at both ages preliminarily. Based on the difference of r between the MZ and DZ twin pairs, the most appropriate model (ACE or ADE) would be selected, and then we calculated variance components based on intraclass correlations [40]. Differences in r between zygosity indicated the differential contribution of genetic and environmental variances of the overall variance in GBI scores, and stratification by age at separation showed its effects, such as modification, on the genetic and environmental variances. Furthermore, a bivariate cross-lagged path model was fitted with separation status and within-pair differences, similarity, in GBI score at both ages with the maximum likelihood estimator (twin pairs were analyzed as the unit of observation). This gauged the structural relationships of repeatedly measured variables and tested both directions between differences and separation, which would could suggest a potential role of genetics [41]. If within-pair difference (instead of at the individual level) leads to separation, genetic effects are suggested, while if separation leads to within-pair difference, we should considered the more environmental roles on the phenotype. Three covariates were adjusted for: sex and zygosity combination (male monozygotic (MMZ), female monozygotic (FMZ), male dizygotic (MDZ), female dizygotic (FDZ), and opposite-sex dizygotic (OSDZ)), paternal education, and maternal education. Twin pairs whose zygosity was unknown or who had missing information on separation status and within-pair differences in GBI score at both ages were excluded from the cross-lagged path model.

Due to the possibility of reverse causality, wherein mental health conditions might influence twins to separate or leave home, we conducted a sensitivity linear regression to examine the association of age at separation with several mental health conditions at age 14, adjusting for sex, zygosity, paternal education, and maternal education. There were five subscales –– depression, social anxiety, hyperactivity, aggression, and inattention –– from the Multidimensional Peer Nomination Inventory (MPNI) was used to assess twins’ mental health at age 14. Each subscale contained a varying number of items (4-point Likert scale), and we used the mean of item responses as the outcome. The MPNI scale has been validated and showed predictive power in the previous study [27]. Due to different instruments to measure depressive symptoms between different ages, depressive symptoms from the MPNI at age 14 were not added to the MMRM. Then, we conducted another sensitivity analysis based on the linear regression for the potential confounding by mental health at age 14. The depression from MPNI was additionally adjusted as a covariate.

The first two hypotheses in the study were tested in the same types of models, but the age at separation and residential mobility were tested independently in models as the exposure of interest. Additionally, we controlled for the cluster effect by twin pair in the regression analyses through “robust” standard errors. Regression coefficients and 95% confidence intervals (CIs) were reported. A two-tailed *P* < 0.05 was considered statistically significant. All statistical analyses were performed using Stata 17.0 (StataCorp, College Station, TX, USA) and Mplus 8.6 (Muthen & Muthen, Los Angeles, CA, USA). The code used in this research can be found at Github (https://github.com/doge73/age-at-separation-GBI).

## Results

### Demographic characteristics

A total of 3071 twins were included in the final analysis including 1284 twin pairs with both twins included and another 503 individual twins (i.e. their cotwin had not participated or otherwise excluded as defined above). As shown in Table 1, the mean score for GBI assessment at age 17 was 5.1, and in young adulthood was 4.4. The majority of twins were female (56.4%), dizygotic (60.6%), and reported never smoking in young adulthood (53.5%). For socioeconomic status, work status, secondary level school, maternal education had significant between-group differences in either or both GBI scores at age 17 and young adulthood. Supplemental Table 1 shows the comparison of some characteristics between individual twins included in the analyses and was available at baseline (*n* = 5104, consented to participate in FinnTwin12 currently), and a similar distribution in both groups suggested a low risk of selection bias.

**Table 1:** Demographic, socioeconomic characteristics, and depressive symptoms for individual twins

Characteristics	Individual n. (%)	Mean (SD) or *P*-value^a^	
		GBI at age 17^b^	GBI in young adulthood^c^
**Overall**	3071	5.1 (4.9)	4.4 (4.68)
**Sex**		< 0.0001	< 0.0001
Male	1339 (43.6)	3.7 (3.9)	3.6 (4.3)
Female	1732 (56.4)	6.2 (5.2)	5.0 (4.9)
**Zygosity**		0.00	0.06
Monozygotic	1066 (34.7)	4.6 (4.6)	4.1 (4.5)
Dizygotic	1861 (60.6)	5.3 (5.0)	4.5 (4.7)
Unknown	144 (4.7)	5.7 (5.2)	4.8 (5.6)
**Smoking**		< 0.0001	< 0.0001
Never	1642 (53.5)	4.4 (4.4)	3.7 (4.1)
Quit	340 (11.1)	6.2 (5.4)	4.7 (5.0)
Occasional	309 (10.1)	5.7 (5.0)	4.5 (4.7)
Current	780 (25.4)	5.9 (5.3)	5.7 (5.4)
**Work**		0.11	< 0.0001
Full-time work	1582 (51.5)	4.9 (4.8)	4.0 (4.3)
Part-time work	394 (12.8)	5.1 (4.4)	4.5 (4.3)
Irregualr work	372 (12.1)	5.1 (4.6)	4.4 (4.4)
Not working	723 (23.5)	5.5 (5.2)	5.3 (5.7)
**Secondary level school**		< 0.0001	< 0.0001
Senior high school	1850 (60.2)	4.9 (4.6)	4.1 (4.4)
Vocational	1042 (33.9)	5.0 (4.8)	4.4 (4.6)
None	179 (5.8)	7.9 (7.2)	7.7 (6.7)
**Paternal education**		0.06	0.39
Missing	496 (16.2)	5.5 (5.4)	4.6 (4.9)
Low	678 (22.1)	5.4 (5.0)	4.5 (4.8)
Medium	1496 (48.7)	4.9 (4.7)	4.3 (4.5)
High	401 (13.1)	5.0 (4.7)	4.5 (4.7)
**Maternal education**		0.00	0.03
Missing	225 (7.3)	6.1 (5.3)	5.4 (6.2)
Low	517 (16.8)	5.7 (5.3)	4.8 (4.9)
Medium	1863 (60.7)	5.0 (4.7)	4.3 (4.5)
High	466 (15.2)	4.7 (4.7)	4.4 (4.8)

^a^*P* value was calculated from the analysis of variance

^b^302 individual twins were missing because they did not provide GBI assessment at age 17

^c^26 individual twins were missing because they did not provide GBI assessment in young adulthood

### Association of age at separation with GBI

The sample sizes of each linear regression analysis are presented in Table 2. After adjustment for covariates, compared to twins separated before age 17, there were significant lower log-transformed GBI scores at age 17 in all three groups of twins, those who separated between ages 17 and 19.5 (coefficient: -0.16, 95% CI: -0.31, -0.02), between ages 19.5 and 22 (coefficient: -0.22, 95% CI: -0.37, -0.08), and after age 22 (coefficient: -0.44, 95% CI: -0.60, -0.28). Moreover, twins who separated between ages 19.5 and 22 and after age 22 also had lower log-transformed GBI scores in young adulthood (coefficient: -0.19, 95% CI: -0.37, -0.02 and coefficient: -0.25, 95% CI: -0.44, -0.06, respectively), compared to twins separated before age 17.

**Table 2:** Association of age at separation and residential mobility with depressive symptoms (GBI) using linear regression

Characteristics	log-transformed GBI score at age 17			log-transformed GBI score in young adulthood			Change of GBI		
	Individual n. (%)	Unadjusted coefficient (95% CI)	Adjusted coefficient (95% CI)^a^	Individual n. (%)	Unadjusted coefficient (95% CI)	Adjusted coefficient (95% CI)^b^	Individual n. (%)	Unadjusted coefficient (95% CI)	Adjusted coefficient (95% CI)^b^
**Age at separation**	2725			2976			2700		
zero to less than 17	110 (4.04)	Ref	Ref	131 (4.40)	Ref	Ref	107 (3.96)	Ref	Ref
17 to less than 19.5	967 (35.49)	-0.18 (-0.34, -0.01)*	-0.16 (-0.31, -0.02)*	1078 (36.22)	-0.21 (-0.40, -0.02)*	-0.17 (-0.35, 0.00)	959 (35.52)	-0.41 (-1.41, 0.60)	-0.22 (-1.19, 0.75)
19.5 to less than 22	1146 (42.06)	-0.34 (-0.50, -0.18)*	-0.22 (-0.37, -0.08)*	1228 (41.26)	-0.31 (-0.50, -0.13)*	-0.19 (-0.37, -0.02)*	1133 (41.96)	-0.05 (-1.03, 0.94)	0.04 (-0.93, 1.00)
22 or more	502 (18.42)	-0.62 (-0.79, -0.44)*	-0.44 (-0.60, -0.28)*	539 (18.11)	-0.43 (-0.63, -0.23)*	-0.25 (-0.44, -0.06)*	501 (18.56)	0.65 (-0.36, 1.66)	0.67 (-0.34, 1.67)
**Number of moves** **before age 17**	2769			3045			2743		
None	736 (26.58)	Ref	Ref	776 (25.48)	Ref	Ref	728 (26.54)	Ref	Ref
Once	733 (26.47)	0.05 (-0.05, 0.15)	0.05 (-0.04, 0.13)	809 (26.57)	0.06 (-0.03, 0.16)	0.06 (-0.03, 0.15)	726 (26.47)	-0.10 (-0.58, 0.38)	-0.07 (-0.55, 0.40)
Twice	549 (19.83)	0.01 (-0.09, 0.12)	-0.00 (-0.10, 0.10)	602 (19.77)	0.12 (0.01, 0.23)*	0.09 (-0.01, 0.19)	544 (19.83)	0.56 (0.04, 1.08)*	0.52 (-0.00, 1.04)
Three times or more	751 (27.12)	0.07 (-0.03, 0.16)	0.00 (-0.09, 0.10)	858 (28.18)	0.15 (0.06, 0.25)*	0.08 (-0.01, 0.18)	745 (27.16)	0.37 (-0.16, 0.89)	0.40 (-0.15, 0.94)
**Total distance of moving (per 100 km) before age 17**	2505	0.01 (-0.02, 0.03)	0.01 (-0.02, 0.03)	2747	0.01 (-0.02, 0.03)	0.01 (-0.02, 0.03)	2482	0.00 (-0.12, 0.12)	0.00 (-0.12, 0.12)

^a^Adjusted for sex, zygosity, smoking, work status, secondary level school, paternal education, and maternal education; for number of moves and total distance of moving before 17, further adjusted for separation before age 17

^b^Adjusted for sex, zygosity, smoking, work status, secondary level school, paternal education, maternal education, and age when twins provided the GBI assessment in young adulthood; for number of moves and total distance of moving before 17, further adjusted for separation before age 17

^*^P < 0.05

**Fig. 1:**
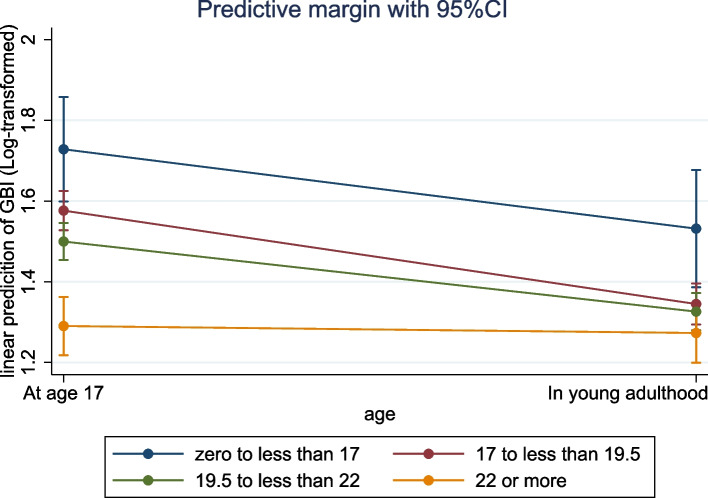
Trajectory of log-transformed GBI score by the age at separation

Figure 1 depicts the trajectory of the predictive marginal mean of log-transformed GBI score from age 17 to young adulthood. The three age-at-separation groups before age 22 decreased in their predictive marginal mean of log-transformed GBI scores, but the latest age at separation group seems not to change notably. The results of the MMRM are presented in Table 3 and included 2712 twins at age 17 and 2962 twins in young adulthood. After controlling for log-transformed GBI score at age 17 and pre-specified covariates, twins who separated between ages 17 and 19.5 (coefficient: -0.19, 95% CI: -0.34, -0.04), between ages 19.5 and 22 (coefficient: -0.21, 95% CI: -0.36, -0.05), and after age 22 (coefficient: -0.26, 95% CI: -0.42, -0.09) scored significantly lower on the GBI (log-transformed) in young adulthood than those who separated before age 17.

**Table 3:** Association of age at separation with depressive symptoms (GBI) using MMRM

Age at separation	Mean (SD)		Adjusted coefficient (95% CI)^a^
	GBI at age 17 (individual n. = 2725)	GB1 in young adulthood (individual n. = 2976)	Log-transformed GBI score in young adulthood
zero to less than 17	6.77 (5.02)	6.20 (5.81)	Ref
17 to less than 19.5	5.91 (5.28)	4.81 (4.89)	-0.19 (-0.34, -0.04)*
19.5 to less than 22	4.91 (4.63)	4.17 (4.37)	-0.21 (-0.36, -0.05)*
22 or more	3.65 (3.98)	3.79 (4.50)	-0.26 (-0.42, -0.09)*

^a^Adjusted for sex, zygosity, smoking, work status, secondary level school, paternal education, maternal education, and age when twins provided the GBI assessment in young adulthood

^*^P < 0.05

Then we performed sex-stratified MMRM of age at separation (Supplemental Table 2). Regardless of the age at separation, males’ mean GBI scores were notably lower than those of females. In females, after controlling for log-transformed GBI score at age 17 and pre-specified covariates, twins who separated after age 22 had lower log-transformed GBI scores in young adulthood (coefficient: -0.29, 95% CI: -0.52, -0.06), compared to twins separated before age 17.

The sensitivity analysis revealed significant correlations of age at separation with depression, hyperactivity, and inattention at age 14 (Supplemental Table 3), suggesting the presence of reverse causality. After further adjusting depression at age 14, compared to those who separated before age 17, twins who separated after age 22 had lower log-transformed GBI scores at age 17 and in young adulthood (Supplemental Table 4). Besides, twins who separated between age 17 and 19.5 also had lower log-transformed GBI scores in young adulthood. After further adjusting depression at age 14, MMRM showed that twins who separated between ages 17 and 19.5 (coefficient: -0.22, 95% CI: -0.42, -0.02), and after age 22 (coefficient: -0.27, 95% CI: -0.50, -0.05) had lower log-transformed GBI scores in young adulthood than those who separated before age 17 (Supplemental Table 5).

### Association of residential mobility with GBI

In the linear regression, after adjustment for covariates, we did not observe any significant association of the number and total distance of moves before age 17 with any of the GBI variables (Table 2). Supplemental Fig. 2 depicts the trajectory of the predictive marginal mean of log-transformed GBI score from age 17 to young adulthood by the number of moves before age 17. The predictive marginal means decreased in all numbers of moves groups and reductions were more substantial in twins who never moved or moved once than in other groups. In MMRM, after controlling for baseline values and covariates, twins who moved twice or moved three or more times had higher GBI (log-transformed) scores in young adulthood than those who never moved (coefficient: 0.10, 95% CI: 0.01, 0.19 and coefficient: 0.09, 95% CI: 0.00, 0.17, respectively) (Supplemental Table 6). After further adjusting depression at age 14, associations attenuated to null (Supplemental Table 4).

### Relationship between separation and within-pair difference

The overall intraclass correlation coefficients of GBI scores among MZ and DZ pairs were 0.56 and 0.14 at age 17 and 0.52 and 0.22 in young adulthood, respectively (Supplemental Table 7). The ADE model was selected (as rMZ > 2rDZ). At age 17, the genetic (A + D) and environmental (E) variances were 0.56 and 0.44, respectively. In young adulthood, the genetic and environmental variances were 0.52 and 0.48, respectively. Notably, for the GBI score in young adulthood, coefficients did not differ between MZs (0.55) compared to DZs (0.53) in twin pairs who separated before age 17.

Supplemental Table 8 presents the distribution of 1280 twin pairs, including in the cross-lagged path model, over the separation status and demographic characteristics. The within-pair difference in GBI scores decreased from age 17 to age 22 overall and in almost every subgroup, except MMZ and FDZ. Within-pair differences in GBI at age 17 were significantly associated with separation status before age 22 (coefficient: 0.01), with a small effect size. Moreover, there were other significant associations between the within-pair differences in GBI score at ages 17 and in young adulthood (coefficient: 0.22) and between separation status before age 17 and age 22 (coefficient: 0.19) (Supplemental Fig. 3).

## Discussion

With a total of 3071 twins from the FinnTwin12 cohort included in this study, we found that later separation in twin pairs was associated with fewer depressive symptoms in later adolescence and young adulthood. In addition, higher residential mobility was linked to more depressive symptoms in young adulthood. Although females had more depressive symptoms than males, there were no obvious sex differences in the association between age at separation and depressive symptoms. However, this assessment was shadowed by reverse causality to some extent, and interpretation and translation should be more cautious. Furthermore, we found a small genetic influence on the separation and fairly minor and inconsistent effects of separation on the difference between cotwins, indicating that age at separation is not a major modifier of sibling difference in depressive symptoms.

Parting from the family of origin is considered as a hallmark of becoming an adult, in which the youth is separated from their parents and siblings, loses their original familial network, and enters a completely new living environment. The majority of the age at separation of the twins in this study was between ages 17 and 22, which also implied life transitions such as entering university to some extent. The transition unavoidably leads to portential negative or positive mental health effects, which are complicated by sibling and familial contexts. Individuals with warm and encouraging siblings relationships may feel small loss, while siblings with relationships marked by conflict may feel mutual relief after separation [42]. Seiffe-Krenke found that the rate of family conflict was higher in adolescents who left home at the age of 21 and 23 years for females and males, respectively, than those still in the nest, and the in-time leaver pattern correlated with a worse level of psychological health [43]. Higher individual and family income were associated with higher odds of home leaving [44, 45], and lower financial stress was mutually correlated with fewer depressive symptoms at the stage of emerging adulthood [46]. With our results, we observed that longer or more family support, indicated by an older age at separation, as well as more mutual support by cotwins, could help adolescents cope with the mental health burden of separation from parents and siblings (same-aged twin sibling in our case) and leaving home. Separation is regarded as a part of the social/family exposome [47]. Extended family support including more relatives has been associated with lower rates of MDD and fewer depressive symptoms [48, 49]. Longer and more support could be extrapolated to better support. Better family involvement and sibling relationships including warmth, attention, and praise from parents predicted less MDD in adulthood and are aspects of corresponding interventions [50–52]. Familial cohesion (emotional bonding) and satisfactory social support were also shown to be helpful in reducing depressive symptoms in lower-middle-class communities in the US [53]. A favorable family background and relationships could be protective factors to support early adulthood transition, and stable transitions were associated with fewer depressive symptoms [54].

Given the evidence for a reverse effect of mental health on age at separation in this study, we further propose a more dynamic effect between family, separation, and mental health. For example, a previous study showed, a later age of leaving was associated with closer residence to aging parents and more frequent contact, compared with siblings who moved out on time, depicting a positive effect from extended coresidence [55]. A UK longitudinal study suggested that parents' relationship quality and early childhood behavioral difficulties were reciprocally linked [56]. Additionally, there was a bidirectional influence of parenting behavior and conduct problems from childhood to adolescence among boys shown in a US study [57]. Furthermore, Stice and Barrera found a full reciprocal relationship between adolescent substance use, but not externalizing, and family support and control [58], while null evidence also exists [59]. On the other hand, age at separation reflected the shared environment between cotwins instead of the separation event itself, thus, to some degree, it also included the familial environment induced by twins’ mental health. This means more complex interactions between individuals, siblings, and families. Future studies require more sophisticated models and a larger number of familial variables, and a lifecourse perspective is recommended.

Residential mobility involves the constant change of the built environment and could be regarded as a part of the physical exposome. Instable residence reduces family interaction, fragments education, cuts social connections, and leads to emotional stress [60]. A solid body of evidence has been established that a strong association between higher childhood residential mobility and poorer mental health at later ages across countries [7, 9, 11]. One study in North Ireland used census-based record linkage [61], while our findings could strengthen this association through longitudinal analysis with rapid updates. Solís et al. developed the biological plausibility that broad early negative experience increased physiological wear-and-tear, measured by allostatic load, which largely explained the health behaviors and education level in later ages [62]. Nevertheless, we should be aware that the effect could be complicated by children’s emotionality, familial context, and reason of moves. Our null results regarding the distance of moves may be due to heterogeneous motivation, wherein a shorter distance could help to maintain the social network and longer distances could be related to work repositioning [7]. Physical moving itself does not necessarily correlate with the separation of family, and company with family is persisting. In a sample of 70 children from rural areas of Georgia, US., children with intense emotions had a surprising reduction in depressive symptoms as a function of increased residential mobility, while children with non-intense emotions experienced the opposite [10]. Additionally, familial support was able to buffer the harmful effect of mobility on children’s education and career achievement [63]. In our study, we had to exclude twins who moved abroad at least once, limiting our analysis by selection bias to some extent. Multiple studies have shown that non-Western immigrant adolescents have a higher risk of mental health problems compared to native European adolescents [64, 65].

Extensive research has investigated the direction between similarity and contact, and it seems that both directions exist, but the magnitude varies by age, sex, and traits [41, 66–68]. Separation is a turning point of contact, since the degree of contact inevitably decreases after separation. Based on the small effect size in the cross-lagged longitudinal analyses, the genetic influence on the separation was minimally observed. Age at separation might modify the similarity of depressive symptoms to a minor degree. The intraclass correlation coefficients were almost always higher in MZs than in DZs at all ages at separation in our study, but the difference decreased in young adulthood and with later separation, showing a slightly decreasing genetic effect. Between ages 17 and 22, the environmental variance increased, which was consistent with increasing E with age reported in a previous study [69]. This was also corroborated by the nearly identical coefficients in the earliest separation group between MZ and DZ in young adulthood. Moreover, as abovementioned, age at separation was reflected as a proxy to the entire familial exposome. Given the heritability of 37% for depressive symptoms in the previous analysis [70], there was vast room for environmental effects including the familial exposome, and our results suggested that genes play a role in the interaction of environmental effects with depressive symptom. A previous twin study in the UK highlighted the genetic influence in shaping a depressogenic environment in middle childhood as a gene–environment correlation [71]. Through DNA methylation, Carreras-Gallo et al. illustrated a gene–environment interaction mechanism through DNA methylation, elucidating its connection with the early-life exposome [72]. Thus, instead of implying any causality, our findings on the role of genetics suggested that age at separation should be considered in refining the assessment of both genetic and environmental influences, revealing the intricate relationships in future studies.

Our study was strengthened by the objective measure of the length that twins stayed in their parental home by age at separation through the national registry, which helps to avoid recall bias and provide more accurate information such as the date of moves. Moreover, in our previous study, the sensitivity and specificity of self-reported separation status were not ideal compared to the age at separation provided through the national system [30]. In addition, the two variables on residential mobility helped us quantify its cumulative and longitudinal effect on the development of twins. Furthermore, the analysis, including repeated measures, could characterize the critical phase of depressive symptom development from late adolescence to young adulthood.

There were also several limitations in this study. First, the sensitivity analysis revealed the probability of reverse causality, suggesting a complex relationship between separation and mental health, especially considering the role of genetics and complex interpersonal network. Although, after adjusting for mid-adolescence depression, our main finding about age at separation was still valid, we should be cautious in interpreting and translating our findings. Second, the 10-item GBI was a non-diagnostic self-report measure of depressive symptoms. However, previous studies have shown high reliability and validity of the full-scale GBI from which this shorter version is derived [73]. As we had interview-based diagnostic information on MDD on the same participants, we could show a high validity of this short GBI. Third, the twin study may lead to the concern of low generalizability or representativeness. Most traits in adult twins including adaptive behavior, depression, and anxiety did not differ from singletons, which were suggested to be generalizable [74–76]. Fourth, when Finnish males are doing their mandatory military service, their residential records do not change to their service location, which may lead to information bias. Additionally, military service could possibly affect mental health [77], which suggests potential roles as a confounder or modifier. Compared to many other countries, the duration of Finnish military service is relatively short, but it is obligatory for all men. Cotwins usually start simultaneously and can spend weekends and holidays together at home. We believe that the influence of military service was neither random nor very substantial, and performed the sex-stratification analysis to investigate this further, but there was no obvious sex-specific effect. Fifth, some potential confounders were not included due to availability such as family wealth or personality. Physical environmental components, such as greenery, as a reflection of social segregation, during adolescence should be also considered in a lifecourse perspective [78]. Although parent’s education could reflect the general family condition to some extent, future access to Finnish social and educational registers may help to address this concern. Finally, the overlap between personality, perhaps some other phenotypes, and depressive symptoms may interfere with the estimation [79], and we would like to further disentangle their complicated relationships with separation in the future.

## Conclusions

Overall, the results provided a novel and valid estimation of the effect of age at separation on depressive symptoms in later adolescence and early adulthood. It could reflect the influence of the length and amount of support from the cotwin and family for twins on addressing this separation challenge and depressive symptoms, however, we should consider a more dynamic relationship between family, separation, and mental health. Moreover, we confirmed the association between residential mobility and mental health with longitudinal analysis. Further intervention targeting the family environment, could reduce the incidence of depressive symptoms.

### Supplementary Information

Supplementary Material 1

## Data Availability

The datasets generated and analysed during the current study are not publicly available due to the restrictions of informed consent but are available from the corresponding author on reasonable request.

## Abbreviations

A: Additive genetic component
AUC: Area under the receiver operating characteristic curve
C: Common environmental component
CI: Confidence intervals
D: Dominant genetic component
DZ: Dizygotic
E: Unique environmental component
FDZ: Female dizygotic
FMZ: Female monozygotic
GBI: General Behavior Inventory
MDZ: Male dizygotic
MMZ: Male monozygotic
MZ: Monozygotic
OSDZ: Opposite-sex dizygotic
r: Intraclass correlation coefficient
