# Supplementary material for: Age at separation, residential mobility, and depressive symptoms among twins in late adolescence and young adulthood: a FinnTwin12 cohort study
Source: BMC Public Health. 2024 Aug 17;24:2239. doi: 10.1186/s12889-024-19734-w (PMC11330072; doi:10.1186/s12889-024-19734-w)
Supplement: Supplementary file 1 — Supplementary Material 1 [file 12889_2024_19734_MOESM1_ESM.docx]

**Title:** Age at separation, residential mobility, and depressive symptoms among twins in late adolescence and young adulthood: a FinnTwin12 cohort study

**Zhiyang Wang et al. – Online supplementary material**

**Content of Supplementary Material**

Supplemental Table 1: Characteristics of individual twins included in analyses or available in baseline

Supplemental Table 2: Sex-stratified association of age at separation with depressive symptoms (GBI) using MMRM

Supplemental Table 3: Association of age at separation with mental health scales measured by the MPNI at age 14

Supplemental Table 4: Sensitivity association of age at separation and residential mobility with depressive symptoms (GBI) using linear regression further adjusted for mid-adolescence depression

Supplemental Table 5: Sensitivity association of age at separation and residential mobility with depressive symptoms (GBI) using MMRM further adjusted for mid-adolescence depression

Supplemental Table 6: Association of number of moves before age 17 with depressive symptoms (GBI) using MMRM

Supplemental Table 7: Intraclass correlation coefficients (r) among MZ and DZ twin pairs for depressive symptoms (GBI)

Supplemental Table 8: Separation status, demographic characteristics, and within-pair differences in depressive symptoms (GBI) score for twin pairs

Supplemental Figure 1: Flowchart of general FinnTwin12 cohort

Supplemental Figure 2: Trajectory of log-transformed GBI score by the number of moves

Supplemental Figure 3: Cross-lagged path model for within-pair differences in depressive symptoms (GBI) and separation status (1080 twin pairs). Numbers over the lines indicate the regression coefficients.

Supplemental Table 1: Characteristics of individual twins included in analyses or available in baseline

| Characteristics | n. (%) / mean (SD) | |
| --- | --- | --- |
|  | Included in analyses | Available in baseline |
| **Overall** | 3071 | 5104 |
| **Sex** |  |  |
| Male | 1339 (43.6) | 2576 (50.5) |
| Female | 1732 (56.4) | 2528 (49.5) |
| **Zygosity** |  |  |
| Monozygotic | 1066 (34.7) | 1646 (32.3) |
| Dizygotic | 1861 (60.6) | 3193 (62.6) |
| Unknown | 144 (4.7) | 265 (5.2) |
| **Body mass index ^a^** | 17.6 (2.6) | 17.6 (2.6) |
| **Paternal education** |  |  |
| Missing | 368 (12.0) | 673 (13.1) |
| Low | 713 (23.2) | 1258 (24.4) |
| Medium | 1573 (51.2) | 2577 (50.1) |
| High | 417 (13.6) | 639 (12.4) |
| **Maternal education** |  |  |
| Missing | 74 (2.4) | 149 (2.9) |
| Low | 551 (17.9) | 1023 (19.9) |
| Medium | 1961 (63.9) | 3237 (62.8) |
| High | 485 (15.8) | 745 (14.5) |

a 118 and 202 individual twins were missing due to body mass index in whose included in analyses and was available in baseline, respectively.

Supplemental Table 2: Sex-stratified association of age at separation with depressive symptoms (GBI) using MMRM

| Age at separation | Mean (SD) | | Adjusted coefficient (95% CI) ^a^ |
| --- | --- | --- | --- |
|  | GBI at age 17 | GB1 in young adulthood | Log-transformed GBI score  in young adulthood |
| *In males (1159 individual twins at age 17 and 1294 individual twins in young adulthood)* | | | |
| zero to less than 17 | 4.89 (4.04) | 4.79 (4.96) | Ref. |
| 17 to less than 19.5 | 3.94 (4.03) | 3.87 (4.62) | -0.20 (-0.42, 0.03) |
| 19.5 to less than 22 | 3.81 (3.99) | 3.41 (3.86) | -0.22 (-0.43, 0.00) |
| 22 or more | 2.86 (3.47) | 3.47 (4.54) | -0.23 (-0.46, 0.00) |
| *In females (1566 individual twins at age 17 and 1682 individual twins in young adulthood)* | | | |
| zero to less than 17 | 8.03 (5.24) | 7.28 (6.21) | Ref. |
| 17 to less than 19.5 | 6.89 (5.55) | 5.31 (4.96) | -0.17 (-0.37, 0.04) |
| 19.5 to less than 22 | 5.86 (4.93) | 4.84 (4.68) | -0.19 (-0.40, 0.02) |
| 22 or more | 4.53 (4.32) | 4.15 (4.44) | -0.29 (-0.52, -0.06)* |

^a^ Adjusted for zygosity, smoking, secondary level school, work, paternal education, maternal education, and age when twins provided the GBI assessment in young adulthood

* *P*<0.05

Supplemental Table 3: Association of age at separation with mental health scales measured by the MPNI at age 14

| Age at separation (n=1175) | Adjusted coefficient (95% CI) ^a^ | | | | |
| --- | --- | --- | --- | --- | --- |
|  | Depression | Anxiety | Hyperactivity | Aggression | Inattention |
| zero to less than 17 | Ref. | Ref. | Ref. | Ref. | Ref. |
| 17 to less than 19.5 | -0.10 (-0.21, 0.01) | -0.12 (-0.27, 0.03) | -0.08 (-0.23, 0.08) | -0.07 (-0.20, 0.06) | -0.11 (-0.28, 0.05) |
| 19.5 to less than 22 | -0.16 (-0.27, -0.06)* | -0.09 (-0.24, 0.06) | -0.12 (-0.27, 0.03) | -0.12 (-0.25, 0.01) | -0.18 (-0.34, -0.02)* |
| 22 or more | -0.16 (-0.28, -0.04)* | -0.17 (-0.34, 0.00) | -0.26 (-0.42, -0.11)* | -0.14 (-0.27, -0.00)* | -0.27 (-0.44, -0.10)* |

^a^ Adjusted for zygosity, smoking, paternal education, and maternal education

* *P*<0.05

Supplemental Table 4: Sensitivity association of age at separation and residential mobility with depressive symptoms (GBI) using linear regression further adjusted for mid-adolescence depression

| Characteristics | log-transformed GBI score at age 17 | | log-transformed GBI score in young adulthood | | Change of GBI | |
| --- | --- | --- | --- | --- | --- | --- |
|  | Individual n. | Adjusted coefficient  (95% CI) ^a^ | Individual n. | Adjusted coefficient  (95% CI) ^b^ | Individual n. | Adjusted coefficient  (95% CI) ^b^ |
| **Age at separation** | 1099 |  | 1187 |  | 1091 |  |
| zero to less than 17 |  | Ref. |  | Ref. |  | Ref. |
| 17 to less than 19.5 |  | -0.14 (-0.33, 0.05) |  | -0.22 (-0.45, -0.00)* |  | -0.16 (-1.6, 1.28) |
| 19.5 to less than 22 |  | -0.18 (-0.37, 0.01) |  | -0.16 (-0.38, 0.07) |  | 0.45 (-0.96, 1.86) |
| 22 or more |  | -0.43 (-0.65, -0.21)* |  | -0.29 (-0.54, -0.04)* |  | 0.59 (-0.93, 2.11) |
| **Number of moves before age 17** | 1111 |  | 1204 |  | 1103 |  |
| None |  | Ref. |  | Ref. |  | Ref. |
| Once |  | 0.12 (-0.02, 0.27) |  | 0.01 (-0.14, 0.15) |  | -0.23 (-1.07, 0.61) |
| Twice |  | 0.05 (-0.11, 0.20) |  | 0.07 (-0.08, 0.23) |  | 0.49 (-0.34, 1.32) |
| Three times or more |  | 0.02 (-0.12, 0.17) |  | -0.03 (-0.17, 0.12) |  | 0.17 (-0.71, 1.05) |
| **Total distance of moving (per 100km) before age 17** | 1004 | 0.02 (-0.01, 0.04) | 1091 | 0.01 (-0.01, 0.04) | 999 | -0.05 (-0.22, 0.12) |

^a^ Adjusted for depression at age 14, sex, zygosity, smoking, work status, secondary level school, and parental education; for number of moves and total distance of moving before 17, further adjusted for separation before age 17

^b^ Adjusted for depression at age 14, sex, zygosity, smoking, work status, secondary level school, parental education, and age when twins provided the GBI assessment in young adulthood; for number of moves and total distance of moving before 17, further adjusted for separation before age 17

Supplemental Table 5: Sensitivity association of age at separation and residential mobility with depressive symptoms (GBI) using MMRM further adjusted for mid-adolescence depression

| Age at separation | Mean (SD) | | Adjusted coefficient (95% CI) ^a^ |
| --- | --- | --- | --- |
|  | GBI at age 17  (individual n.=1099) | GB1 in young adulthood (individual n.=) | Log-transformed GBI score  in young adulthood |
| zero to less than 17 | 6.58 (4.39) | 6.50 (4.10) | Ref. |
| 17 to less than 19.5 | 5.89 (5.46) | 5.90 (5.48) | -0.22 (-0.42, -0.02)* |
| 19.5 to less than 22 | 4.74 (4.79) | 4.74 (4.80) | -0.16 (-0.36, 0.05) |
| 22 or more | 3.77 (4.37) | 3.78 (4.39) | -0.27 (-0.50, -0.05)* |

^a^ Adjusted for depression at age 14, sex, zygosity, smoking, work status, secondary level school, paternal education, maternal education, and age when twins provided the GBI assessment in young adulthood

* *P*<0.05

Supplemental Table 6: Association of number of moves before age 17 with depressive symptoms (GBI) using MMRM

| Number of moves before age 17 | Mean (SD) | | Adjusted coefficient (95% CI) ^a^ |
| --- | --- | --- | --- |
|  | GBI score at age 17  (individual n =2769) | GB1 score in young adulthood  (individual n=3045) | Log-transformed GBI score  in young adulthood |
| None | 4.87 (4.60) | 3.94 (4.20) | Ref. |
| Once | 5.19 (5.00) | 4.25 (4.52) | 0.06 (-0.02, 0.14) |
| Twice | 4.95 (4.69) | 4.70 (5.10) | 0.10 (0.01, 0.19)* |
| Three times or more | 5.32 (5.06) | 4.78 (4.89) | 0.09 (0.00, 0.17)* |

^a^ Adjusted for sex, zygosity, smoking, secondary level school, work, paternal education, maternal education, separation before age 17, and age when twins provided the GBI assessment in young adulthood

* *P*<0.05

Supplemental Table 7: Intraclass correlation coefficients (r) among MZ and DZ twin pairs for depressive symptoms (GBI)

| Intraclass correlation (Total 1284 twin pair) | GBI score at age 17 ^a^ | | GBI score in young adulthood ^b^ | |
| --- | --- | --- | --- | --- |
|  | MZ twin pair r  (n. twin pairs) | DZ twin pair r  (n. twin pairs) | MZ twin pair r  (n. twin pairs) | DZ twin pair r  (n. twin pairs) |
| **Overall** | 0.56 (434) | 0.14 (680) | 0.52 (469) | 0.22 (739) |
| **Age at separation ^c^** |  |  |  |  |
| zero to less than 17 | 0.63 (16) | 0.14 (23) | 0.55 (20) | 0.53 (28) |
| 17 to less than 19.5 | 0.64 (139) | 0.14 (257) | 0.58 (155) | 0.14 (280) |
| 19.5 to less than 22 | 0.46 (180) | 0.06 (279) | 0.51 (188) | 0.19 (301) |
| 22 or more | 0.50 (86) | 0.16 (118) | 0.40 (92) | 0.33 (127) |

^a^ 170 twin pairs were missing due to unknown zygosity or no GBI score in at least one cotwin at age 17

^b^ 75 twin pairs were missing due to unknown zygosity or no GBI score in at least one cotwin in young adulthood

^c^ Additional 16 and 17 twin pairs were missing due to age at separation for GBI score at age 17 and in young adulthood, respectively

Supplemental Table 8: Separation status, demographic characteristics, and within-pair differences in depressive symptoms (GBI) score for twin pairs

| Characteristics | Twin pair n. (%) | Mean (SD) | |
| --- | --- | --- | --- |
|  |  | Within-pair difference of GBI score | |
|  |  | At age 17 | In young adulthood |
| **Included in cross-lagged panel model** | 1080 | 3.9 (4.1) | 3.6 (3.7) |
| **Separation before age 17** |  |  |  |
| No | 1043 (96.6) | 3.9 (4.1) | 3.6 (3.7) |
| Yes | 37 (3.4) | 4.4 (3.8) | 3.6 (4.2) |
| **Separation before age 22** |  |  |  |
| No | 203 (18.8) | 3.1 (3.6) | 3.1 (3.3) |
| Yes | 877 (81.2) | 4.2 (4.2) | 3.7 (3.8) |
| **Sex and zygosity combination** |  |  |  |
| MMZ | 163 (15.1) | 2.4 (2.8) | 2.4 (2.8) |
| FMZ | 251 (23.2) | 3.2 (3.0) | 3.1 (3.1) |
| MDZ | 144 (13.3) | 3.8 (3.8) | 3.3 (3.8) |
| FDZ | 206 (19.1) | 4.5 (4.3) | 4.5 (4.3) |
| OSDZ | 316 (29.3) | 5.0 (4.9) | 4.1 (4.0) |
| **Paternal education** |  |  |  |
| Missing | 110 (10.2) | 4.6 (5.5) | 3.9 (3.7) |
| Low | 253 (23.4) | 4.0 (4.0) | 3.7 (3.9) |
| Medium | 564 (52.2) | 3.7 (3.8) | 3.4 (3.6) |
| High | 153 (14.2) | 4.2 (4.2) | 3.9 (4.2) |
| **Maternal education** |  |  |  |
| Missing | 27 (2.5) | 6.4 (4.8) | 4.2 (3.0) |
| Low | 183 (16.9) | 4.4 (4.6) | 3.6 (3.6) |
| Medium | 690 (63.9) | 3.7 (3.8) | 3.6 (3.8) |
| High | 180 (16.7) | 3.9 (4.2) | 3.6 (3.9) |

Supplemental Figure 1: Flowchart of general FinnTwin12 cohort


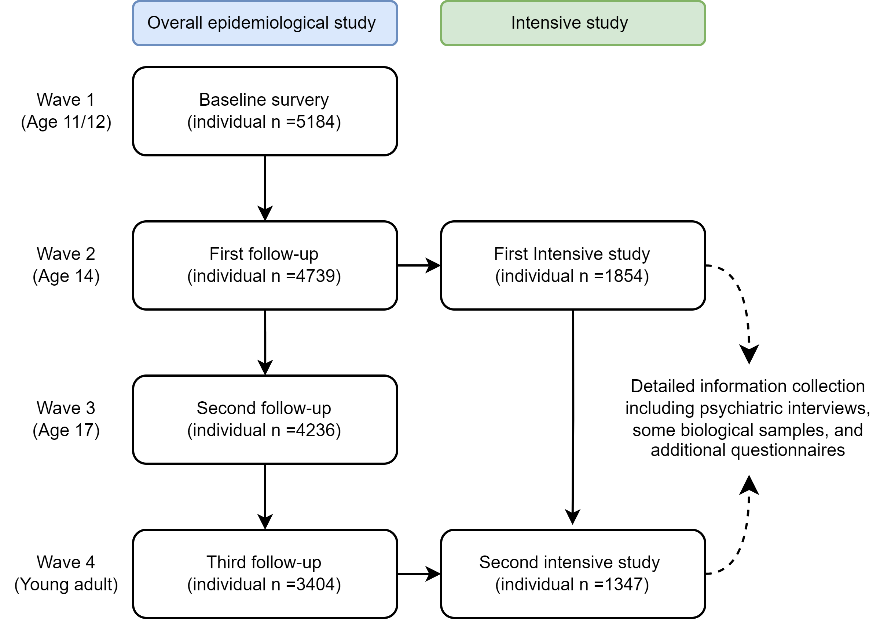


Supplemental Figure 2: Trajectory of log-transformed GBI score by the number of moves

Supplemental Figure 3: Cross-lagged path model for within-pair differences in depressive symptoms (GBI) and separation status (1080 twin pairs). Numbers over the lines indicate the regression coefficients.


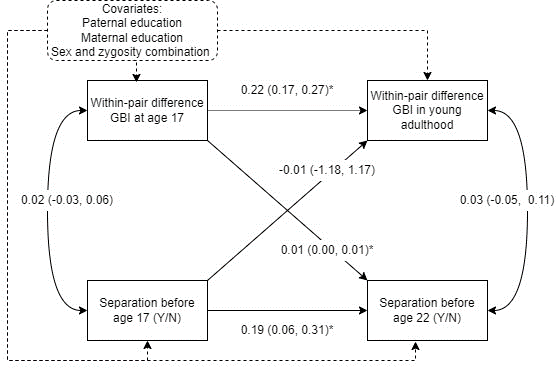


* *P*<0.05

Note: Numbers over the lines indicate the regression coefficients (95% CI).
